# Dysregulated Microglial Cell Activation and Proliferation Following Repeated Antigen Stimulation

**DOI:** 10.3389/fncel.2021.686340

**Published:** 2021-08-10

**Authors:** Sujata Prasad, Wen S. Sheng, Shuxian Hu, Priyanka Chauhan, James R. Lokensgard

**Affiliations:** Neurovirology Laboratory, Department of Medicine, University of Minnesota, Minneapolis, MN, United States

**Keywords:** viral reactivation, microglia, neurotoxicity, reactive gliosis, resident memory T-cells, immune activation

## Abstract

Upon reactivation of quiescent neurotropic viruses antigen (Ag)-specific brain resident-memory CD8+ T-cells (bT_RM_) may respond to *de novo*-produced viral Ag through the rapid release of IFN-γ, which drives subsequent interferon-stimulated gene expression in surrounding microglia. Through this mechanism, a small number of adaptive bT_RM_ may amplify responses to viral reactivation leading to an organ-wide innate protective state. Over time, this brain-wide innate immune activation likely has cumulative neurotoxic and neurocognitive consequences. We have previously shown that HIV-1 p24 Ag-specific bT_RM_ persist within the murine brain using a heterologous prime-CNS boost strategy. In response to Ag restimulation, these bT_RM_ display rapid and robust recall responses, which subsequently activate glial cells. In this study, we hypothesized that repeated challenges to viral antigen (Ag) (modeling repeated episodes of viral reactivation) culminate in prolonged reactive gliosis and exacerbated neurotoxicity. To address this question, mice were first immunized with adenovirus vectors expressing the HIV p24 capsid protein, followed by a CNS-boost using Pr55Gag/Env virus-like particles (HIV-VLPs). Following the establishment of the bT_RM_ population [>30 days (d)], prime-CNS boost animals were then subjected to *in vivo* challenge, as well as re-challenge (at 14 d post-challenge), using the immunodominant HIV-1 AI9 CD8+ T-cell epitope peptide. In these studies, Ag re-challenge resulted in prolonged expression of microglial activation markers and an increased proliferative response, longer than the challenge group. This continued expression of MHCII and PD-L1 (activation markers), as well as Ki67 (proliferative marker), was observed at 7, 14, and 30 days post-AI9 re-challenge. Additionally, *in vivo* re-challenge resulted in continued production of inducible nitric oxide synthase (iNOS) with elevated levels observed at 7, 14 and 30 days post re-challenge. Interestingly, iNOS expression was significantly lower among challenged animals when compared to re-challenged groups. Furthermore, *in vivo* specific Ag re-challenge produced lower levels of arginase (Arg)-1 when compared with the challenged group. Taken together, these results indicate that repeated Ag-specific stimulation of adaptive immune responses leads to cumulative dysregulated microglial cell activation.

## Introduction

Inflammatory responses are beneficial when activated in a properly regulated manner for a defined period; however, prolonged or excessive dysregulated inflammation causes numerous chronic, systemic diseases such as arthritis, multiple sclerosis, systemic lupus erythematosus, and many others (Amor et al., 2014; Straub and Schradin, 2016). In spite of the fact that the central nervous system (CNS) poses a unique microenvironment, which normally restricts immune responses, it does exhibit key features of inflammation and peripheral immune cells are recruited in response to brain infections. Inflammatory reactions following viral infection are dominated by MHC class I-restricted CD8+ T lymphocytes (Mutnal et al., 2011; Kim and Shin, 2019). These cells are key players in adaptive immune responses, which restrict viral spread through cytotoxic responses, as well as other associated inflammatory events. Persisting T lymphocytes have been detected within postmortem brain tissue following viral infection and have led to the investigations of T-cell: microglial cell interactions (Smolders et al., 2018; Williams et al., 2020). Because the largely non-regenerating brain is more sensitive to cellular injury, we hypothesized that repeated challenges to viral antigen (Ag) (modeling repeated episodes of viral reactivation) culminate in prolonged reactive gliosis and defective microglial cell functions.

Pathogenic T-cells induce inflammation upon recognition of cognate Ag and are known to contribute to tissue damage during both systemic and localized infection (Steinbach et al., 2018). These Ag-specific cells dramatically change their behavior and their interaction with resident immune cells within the brain when they encounter cognate Ag (Prasad et al., 2018, 2019; Prasad and Lokensgard, 2019). Pathogenic T-cell activation-induced inflammation has been reported in CNS autoimmune disease (Fletcher et al., 2010; Ji et al., 2010). Moreover, various elegant studies using multiple mouse models have demonstrated increased T-cell infiltration during chronic neurodegeneration (Wakim et al., 2010, 2012; Prasad et al., 2015). A population of memory T-cells residing within the brain plays a major role in shaping neuropathological outcomes (Smolders et al., 2018; Wu et al., 2018; Mockus et al., 2019).

Tissue-resident memory T-cells (T_RM_) are a population of T lymphocytes that reside long-term within peripheral tissues. Like other tissues, it has been demonstrated that Ag-specific T_RM_ cells reside within the brain (bT_RM_; Wakim et al., 2010, 2012; Park and Kupper, 2015; Prasad et al., 2017). These cells survey for their cognate Ag and are able to initiate immediate, enhanced, tissue-wide inflammation for pathogen removal in the case of re-infection or reactivation. We and others have reported that these Ag-specific T_RM_ cells respond quickly to low amounts of Ag through abundant cytokine production, such as IFN-γ, IL-2, TNF-α, and other cytotoxic mediators (Cox et al., 2013; Steinbach et al., 2018; Tan et al., 2018; Prasad et al., 2019). These proinflammatory cytokines, particularly IFN-γ, are well known to produce chronic immune activation (Mutnal et al., 2011; Lokensgard et al., 2015; Ta et al., 2019).

Pathogenic stimuli and proinflammatory cytokines produced by Ag-specific bT_RM_ trigger microglial cell activation. It has been well documented that mediators like IFN-γ, TNF-α, and IL-1β amplify the activation of resident glia (Schachtele et al., 2014; Bachiller et al., 2018; Prasad et al., 2019). In particular, IFN-γ production by T lymphocytes serves as an activating factor for microglia and evokes an exaggerated response to secondary inflammatory stimuli (Prasad et al., 2019). Activated microglia subsequently influence the surrounding brain microenvironment through the production of various pro-inflammatory (e.g., IL-1, IL-6, IL-12, TNF-α) and anti- inflammatory cytokines (e.g., TGF-β, IL-10) as well as neurotoxic mediators such as inducible nitric oxide synthase (iNOS; Hanisch, 2002; Wang et al., 2015; Lively and Schlichter, 2018). IFN-γ, together with TNF-α, triggers nitric oxide-induced neurodegeneration (Belkhelfa et al., 2014). Thus, while microglial activation is critical to clear invading pathogens, inflammatory imbalances may impact their function and contribute to neurotoxicity and associated neurodegeneration.

Brain tissues are well-known reservoirs of latent viral infection, including herpesviruses as well as HIV-1, and there is considerable evidence that reactivation occurs frequently under various disease settings (Luzuriaga et al., 2015; Doll et al., 2020; Forte et al., 2020; Marcocci et al., 2020). Although reactivation of these latent infections may result in recurrent disease over time, well-regulated sequences of cellular events normally generate rapid and effective host defense against the reactivated pathogens (Tan et al., 2018; Prasad et al., 2019). Through *de novo* production of foreign viral Ag, reactivation has the potential to exaggerate adaptive memory responses and mounting evidence indicates that even small amounts of Ag may cause extensive collateral tissue damage and neuronal dysfunction (Doll et al., 2020; Marcocci et al., 2020). However, cellular events involved in clearing reactivation-produced Ag remain to be elucidated.

Recent findings from our laboratory demonstrate that challenges of bT_RM_ to specific epitope peptides induce activation of microglia (Prasad et al., 2019). To study the impact of repeated Ag stimulation on microglial cell activation and its associated neurotoxicity, in a scenario modeling latent viral brain infection and its intermittent reactivation, we used our well-established prime-CNS boost model. Using this model, we have previously demonstrated that viral Ag triggered subsequent adaptive responses. This study is a follow-up of our previous findings. Here, we provide evidence that repeated Ag-specific stimulation of adaptive immune responses from bT_RM_ leads to cumulative dysregulated microglial cell activation, proliferation, and neurotoxic mediator production.

## Materials and Methods

### Ethical Statement

This study was carried out in strict accordance with recommendations in the Guide for the Care and Use of Laboratory Animals of the National Institutes of Health. The protocol was approved by the Institutional Animal Care and Use Committee (Protocol ID #: 2001-37808A) of the University of Minnesota. Surgery was performed under Ketamine/Xylazine anesthesia and all efforts were made to minimize suffering. Following surgery, all animals were routinely cared for in accordance with RAR (Research Animal Resources) before being euthanized using isoflurane. Institutional Biosafety Committee (IBC) guidelines and recommended procedures were strictly followed (Protocol ID #: 2005-38144H).

### Virus and Animals

Production of a 2nd-generation (i.e., ΔE1 + ΔE3), replication-incompetent adenovirus vector which expresses the HIV-1 capsid protein p24 under control of the minimal CMV IE promoter (i.e., rAd5-p24) was outsourced to Cyagen Biosciences (Santa Clara, CA, USA). Female BALB/c mice (6–8 week old) were infected with rAd5-p24 (1 × 10^10^ PFU/mouse) *via* tail-vein injection. To promote immune cell infiltration and retention within the brain, animals were then boosted [day (d) 7 later] *via* an intracranial injection of HIV virus-like particles (HIV-VLPs). Animals received 300 fluorescent units (FU) of HIV-VLP in a volume no greater than 5 μl delivered into striatum *via* stereotaxic injection.

### Production of HIV Virus-Like Particles (HIV-VLPs)

Production of HIV-VLPs was performed by transfection of HEK 293T cells with pEYFP-N3 HIV-1, a Gag-expressing codon-optimized plasmid encoding the 55 kDa Gag precursor protein fused to an enhanced yellow fluorescent protein (EYFP), under control of the human CMV IE promoter, obtained from Louis Mansky (Institute for Molecular Virology, University of Minnesota); p3NL(ADA) env, encoding a full-length R5-tropic envelope protein, under control of the HIV-1 LTR promoter, was obtained with permission from Eric Freed (NCI, Frederick, MD, USA). Because these HIV-VLPs express EYFP, FU were used as an indicator of quantity and were quantified using a Spectramax M2 Fluorescent reader 485ex/538em at 530 nm cut-off. An HIV-VLP dose of 300 FU equivalents conferred reliable stimulation and promoted peripheral immune cell infiltration into the brain.

### Intracranial Injection of Mice

Intracranial injection of mice was performed as previously described with slight modifications (Cheeran et al., 2007). Briefly, 6–8 week old female animals were anesthetized using a combination of Ketamine and Xylazine (100 mg and 10 mg/kg body weight, respectively) and immobilized on a small animal stereotactic instrument equipped with a Cunningham mouse adapter (Stoelting Co., Wood Dale, IL, USA). To minimize pain, subcutaneous injection of the analgesic bupivacaine [1–2 mg/kg (0.4–0.8 ml/kg of a 0.25% solution), Hospira, Inc., Lake forest, IL, USA] prior to incision was performed. The skin and underlying connective tissue were reflected to expose reference sutures (sagittal and coronal) on the skull. The sagittal plane was adjusted in a manner that Bregma and lambda were positioned at the same coordinates on the vertical plane. A burr hole was drilled to expose the underlying dura at pre-determined coordinates to access the left striatum (AP = 0 mm, ML = 2.0 mm from Bregma, and DV = 3.0 mm from skull surface). Animals received 300 FU of HIV-VLP in a volume no greater than 5 μl delivered to the striatum *via* stereotaxic injection, using a Hamilton syringe (10 μl) fitted to a 27 G needle. The injection was delivered 1 μl/min over a period of 5 min. The needle was retracted slowly and the burr hole was closed using sterile bone-wax. The animals were then removed from the stereotaxic apparatus, placed on a heating pad and the skin incision was closed with 4-0 silk sutures with an FS-2 needle (Ethicon, Somerville, NJ, USA).

### Ag Challenge and Re-challenge

Previous studies have identified the capsid p24 H-2K^d^ MHC class I-restricted peptide AMQMLKETI (i.e., AI9) as an immunodominant epitope (Tan et al., 2016, 2018). *In vivo* challenge (i.e., first AI9 injection) and re-challenge (i.e., second AI9 injection) were performed by injecting animals with HIV-1-specific T-cell epitope peptide AI9. Ag-challenge was performed d 30 post-HIV-VLP administration, whereas re-challenge was performed at d 14 post-challenge. Animals were injected with 100 μM AI9 peptide in 5 μl saline delivered into the brain striatum. Brain tissue was isolated at d 2, 7, 14, and 30 post-challenge and re-challenge. Brain-derived mononuclear cells (BMNC) were examined for Ag-specific CD8+ T-cells, as well as microglial cell activation, by flow cytometry.

### Brain Leukocyte Isolation and Flow Cytometry Analysis

BMNC were isolated from brains of prime-CNS boost animals using a previously described procedure with minor modifications (Ford et al., 1995; Marten et al., 2003; Cheeran et al., 2007; Mutnal et al., 2011). Briefly, whole brain tissues were harvested (*n* = 6 animals/group/experiment) without enzymatic treatment, and minced finely using a scalpel, and the minced tissues were stirred on a magnetic stirrer for 30 min in RPMI 1640 (2 g/L D-glucose and 10 mM HEPES). Single cell preparations of infected brains were resuspended in 30% Percoll (Sigma-Aldrich, St. Louis, MO) and banded on a 70% Percoll cushion at 900× *g* for 30 min at 15°C. Brain leukocytes obtained from the 30–70% Percoll interface were collected. Cells were treated with Fc block (anti-CD32/CD16 in the form of 2.4G2 hybridoma culture supernatant with 2% normal rat and 2% normal mouse serum) to inhibit non-specific Ab binding. Cells were then counted using the trypan blue dye exclusion method, and 1 × 10^6^ cells were subsequently stained using anti-mouse immune cell surface markers for 15–20 min at 4°C (anti-CD45-BV605, anti-PD-L1-PE-Cy7, anti-CD11b-AF700, anti-CD103-FITC, anti-CD69-eF450, anti-MHCII-APC (Thermo Fisher Scientific, Waltham, MA, USA) and anti-CD8-BV-510 from (Biolegend, San Diego, CA, USA). Control isotype Abs were used for all fluorochrome combinations to assess nonspecific Ab binding. For tetramer staining, the H-2K^d^ MHC class I-restricted peptide AI9 tetramer was purchased from MBL Corporation (Woburn, MA, USA) and used for the evaluation of Ag-specific responses. Samples were acquired using an LSRII H4760 (BD Biosciences, San Jose, CA, USA) flow cytometer, whereas others were acquired using Fortessa X-20 (BD Biosciences). Live leukocytes were gated using forward scatter and side scatter parameters and data were analyzed using FlowJo software (FlowJo, Ashland, OR, USA).

### Intracellular Cytokine Staining

To assess the production of iNOS and Arg1, and to evaluate the proliferative response of the microglial cells, intracellular staining was performed. BMNC (2 × 10^6^ cells/well) were surface stained prior to fixation/permeabilization using cytofix/cytosperm kit (eBioscience, now Thermo Fisher Scientific). Cells were then stained using iNOS-PE, Ki67-PE-cy7 (Thermo Fisher Scientific), and Arg1-FITC (R&D Systems, Minneapolis, MN, USA) as recommended by the manufacturer’s protocol. Stained cells were analyzed using flow cytometry as described above.

### Primary Microglial Cell Cultures

Murine cerebral cortical cells were collected from neonatal Balb/c mice (1 d old). These cells were dissociated after a 30 min trypsinization (0.25%) in HBSS and plated in 75 cm^2^ culture flasks in DMEM containing 6% FBS, penicillin (100 U/ml), streptomycin (100 μg/ml), gentamicin (50 μg/ml), and Fungizone^®^ amphotericin B (250 pg/ml). The medium was replenished at d 1 and 4 after plating. On d 12 of culture, floating microglial cells were harvested and plated onto 48-well cell culture plates (1 × 10^5^ cells/well). After a 1 h incubation at 37°C, the culture plates were washed and incubated overnight before starting the experiments. Purified microglial cell cultures stained positively with Iba-1 antibodies (>95%) and 3–5% with glial fibrillary acid protein (GFAP) antibodies, a specific astrocyte marker.

### Real-Time RT-PCR

For real-time RT-PCR, BMNC collected from animals at d 14 post-challenge were either stimulated with AI9 peptide or left unstimulated for 1 h before being co-cultured with purified microglial cell (>95% microglia) for 24 h. Total RNA was extracted from co-cultured cells using the RNeasy^®^ Lipid Tissue Mini kit (Qiagen, Valencia, CA, USA), treated with DNase and reverse transcribed to cDNA with oligo (dT)_12–18_, random hexmer, dNTPs (Gene Link, Hawthorne, NY), RNase inhibitor and SuperScript™ III reverse transcriptase (Invitrogen, Carlsbad, CA, USA). Diluted cDNA, primers, and SYBR^®^ Advantage^®^ qPCR premix (Takara Bio USA, Mountain View, CA, USA) were subjected to real-time PCR (Bio-Rad Laboratories, Hercules, CA, USA) according to the manufacturer’s guidelines. Primer sequences used were: sense 5′-GCGTCATTGAATCACACCTG-3′ and antisense 5′-GACCTGTGGGTTGTTGACCT-3′ for IFN-γ (104 bp); sense 5′-TGGCCACCTTGTTCAGCTACG-3′ and antisense 5′-GCCAAGGCCAAACACAGCATA-3′ for iNOS (213 bp); sense 5′-AGACTTCCATCCAGTTGCCTTC-3′ and anti-sense 5′-CATTTCCACGATTTCCCAGAG-3′ for IL-6 (175 bp) ; sense 5′-CTGTGAAGGGAATGGGTGTT-3′ and antisense 5′-GGTCACTGTCCCAGCATCTT-3′ for TNF-α (200 bp); sense 5′-CGTGAGTGGGAAGAGAAGTGTC-3′ and antisense 5′-CTACAATGAGGAACAACAGGATGG-3′ for PD-L1 (239 bp) and sense 5′-TGCTCGAGATGTCATGAAGG-3′ and antisense 5′-AATCCAGCAGGTCAGCAAAG-3′ for HPRT (hypoxanthine phosphoribosyltransferase, 95 bp). The PCR conditions for the Bio-Rad CFX96 qPCR System were: 1 denaturation cycle at 95°C for 10 s; 40 amplification cycles of 95°C for 10 s, 60°C annealing for 10 s, and elongation at 72°C for 10 s; followed by 1 dissociation cycle. The relative product levels were quantified using the 2^−ΔΔCt^ method (Livak and Schmittgen, 2001) and were normalized to the housekeeping gene HPRT.

### Statistical Analysis

Data are representative of two separate experiments, each experiment using six animals per group per time point, where brain tissue homogenates of two animals were pooled together. The results were analyzed by the student’s *t*-test as well as One-way ANOVA using GraphPad prism 7.03 software. A value of *p* < 0.05 was considered significant.

## Results

### Ag-Specific CD69+CD103+CD8+ bT_RM_ Cells Persist and Proliferate in Response to Re-challenge

**Figure 1:**
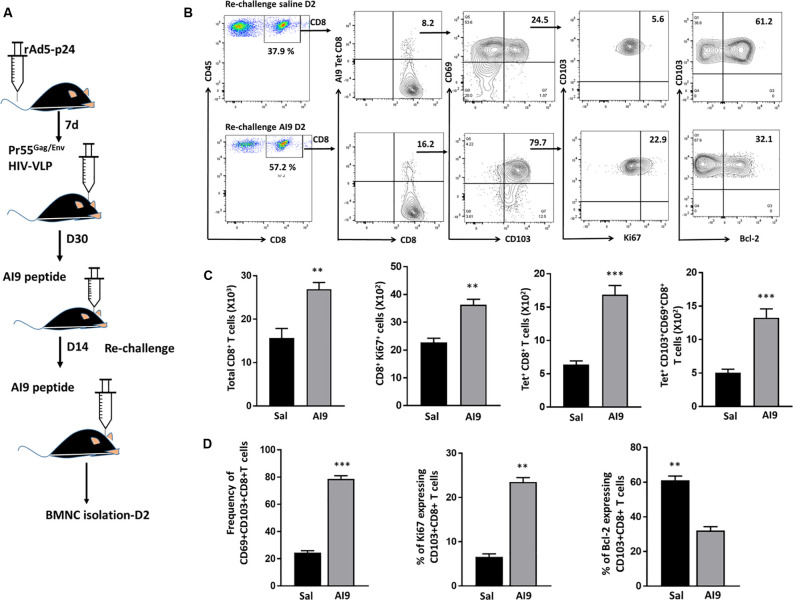
Ag-specific CD69+CD103+CD8+ brain resident-memory T-cells persisted and proliferated in response to re-challenge. **(A)** Schematic of the experimental design illustrates intravenous delivery of recombinant adenovirus vectors expressing HIV-1 p24 capsid protein (rAd5-p24), followed by a CNS-boost consisting of intracranial injection of HIV-VLPs into the striatum. Thirty days later, *in vivo* challenge was performed by injecting cognate antigen (AI9 peptide) or saline (controls) into the brain. Subsequent re-challenge was performed at d 14 post-challenge. BMNC were collected at d 2 post re-challenge. **(B)** Representative flow cytometry plots show the expression of CD8+ T-cells, tetramer-specific CD8+ T-cells, tetramer-specific CD69+CD103+CD8+ bT_RM,_ Ki67 (i.e., proliferating cells) as well as Bcl-2 (i.e., survival marker) expression on tetramer-specific CD69+CD103+CD8+ bT_RM_ at d 2 post-AI9 re-challenge (bottom panel of contour plot), and saline control (upper panel). **(C)** Absolute numbers of brain infiltrating CD8+ T-cells, proliferating CD8+ T-cells, tetramer-specific CD8+ T-cells, and Ag-specific CD69+CD103+CD8+ bT_RM_ were determined at d 2-post re-challenge. **(D)** Pooled data present the frequencies (mean ± SD) of Ag-specific CD69+CD103+CD8+ bT_RM_, percent proliferating, and percent expressing Bcl-2 among the re-challenge group at d 2. Data presented are representative of two separate experiments, each experiment used six animals per group per time point with brain tissue homogenates from two animals being pooled. **p < 0.01 and ***p < 0.001.

In our previous study, we demonstrated that the bT_RM_ cells persist within the murine brain and that their subsequent Ag-specific challenge induced reactive gliosis. In this study, we followed-up on those findings by first demonstrating that bT_RM_ proliferate in response to re-challenge with the immunodominant HIV-1 AI9 CD8+ T-cell epitope peptide. To achieve this, BALB/c mice were first immunized *via* tail vein injection with recombinant adenovirus vectors expressing the HIV-1 p24 capsid protein (rAd5-p24), followed by a CNS-boost using Pr55Gag/Env virus-like particles (HIV-VLPs) injected into the striatum. Following the establishment of the bT_RM_ population (>30 d), which we have previously characterized and reported (Prasad et al., 2019), prime-CNS boost animals were then subjected to *in vivo* Ag-challenge (d 30 post HIV-VLP injection), as well as re-challenge (d 14 post-challenge), using the AI9 CD8+ T-cell epitope peptide (Figure 1A). Knowing that T_RM_ cells rapidly respond to small amounts of Ag, we went on to investigate the total CD8+ T-cell population, Ki67+CD8+ T-cells, Ag-specific CD8+ T-cells, and Ag-specific CD103+CD69+CD8+ T-cells in response to Ag. The re-challenged group of animals displayed an elevated total number of CD8+ T-cells and the difference was found to be significant when compared to saline controls. Moreover, Ki67 expression on total CD8+ T-cells revealed that a sizable fraction readily proliferated seeing its antigen. These total CD8+ T-cells were then analyzed for the absolute number of Ag-specific CD8+ cells. Our data revealed significantly increased levels of tetramer-specific CD8 T-cells among the re-challenged group. Similar observations were made when we analyzed the number of Ag-specific CD103+CD69+CD8+ T-cells (Figures 1B,C). In addition to the absolute numbers, we analyzed the frequencies of these populations. We found that the majority of AI9 tetramer-specific CD8+ T-cells co-expressed CD69 and CD103 at d 2 post re-challenge. The frequency of Ag-specific CD69+CD103+CD8+ T-cells was significantly higher in the AI9 re-challenged group (78.7 ± 1.1%) than the saline group (24.4 ± 0.7%) at d 2 post re-challenge (Figures 1B,D). We next evaluated the proliferative response and survival of the bT_RM_ cells following re-challenge. It was interesting to note that Ag-specific CD69+CD103+CD8+ T-cells displayed increased proliferation following repeated exposure to specific peptide, however, these cells expressed lower levels of Bcl-2 (a marker of cell survival; Figures 1B,D). In sharp contrast, Ag-specific bT_RM_ cells in the saline group showed significantly lower levels of proliferation, whereas their survival was significantly higher when compared to the Ag-specific re-challenge animals. Additionally, bT_RM_ cells in the AI9 re-challenged group showed significantly higher expression of Ki67 (marker for proliferation) 23.5 ± 0.98% than those in the saline group which expressed about 6.5 ± 0.7% Ki67. We also found elevated levels of Bcl-2 expression (61 ± 2.4%) on bT_RM_ cells in the saline group whereas, the AI9 re-challenged group expressed about 32 ± 2.3% of Bcl-2 which was significantly lower (Figures 1B,D). In addition, we analyzed the Ag-specific CD103- population among the saline group of animals and observed lower expression of Bcl-2, which may indicate reduced survival of these CD103- cells (Supplementary Figure 2D).

### Prolonged Microglial Activation Following *In vivo* Re-challenge With T-Cell Epitope Peptides

**Figure 2:**
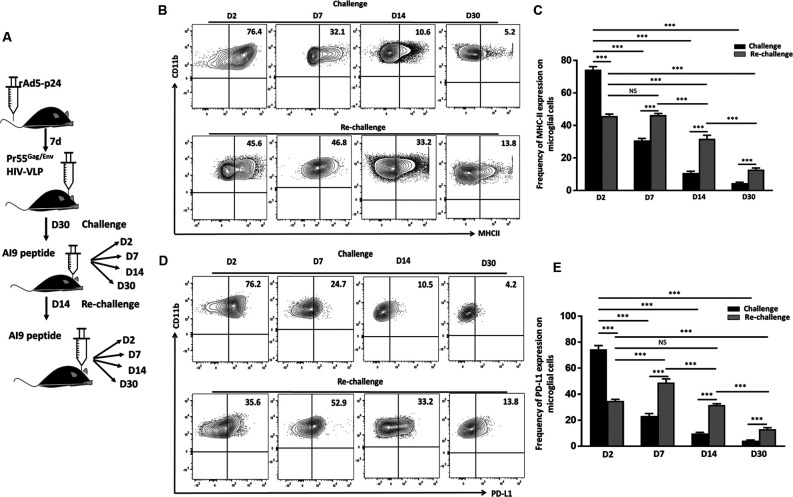
*In vivo* re-challenge with T-cell epitope peptide-induced long-term microglial cell activation. **(A)** Schematic design of AI9 peptide injection into the brain and tissue collection at d 2, 7, 14, and 30 post *in vivo* challenge and re-challenge. **(B)** Contour plots present MHCII expression on CD45^int^/CD11b^+^ microglial cells at the indicated time points post-challenge and re-challenge. **(C)** Bar graph presents the pooled frequency (as a percentage) of MHCII expression on microglia at d 2, d 7, d 14, and d 30 post-challenge and re-challenge. **(D)** Representative contour plots present expression of PD-L1 on microglia at d 2, 7, 14, and 30 post-challenge and re-challenge. **(E)** Bar graph presenting pooled data of the percentage of PD-L1 expression on microglial cells. Data presented are representative of two independent experiments using six animals/group/time point (i.e., two brains pooled together). *P* values were derived from one-way ANOVA statistical testing. ***p < 0.001; non-significant (NS) represent groups with no significant differences.

We have previously reported an immediate and dramatic upregulation of microglial activation markers in response to *in vivo* exposure of specific epitope peptides (Prasad et al., 2019). To investigate the impact of repeated Ag challenge on these resident immune cells of the brain, we examined the effects of challenge and re-challenge. First, we examined the activation of microglia (CD45^int^/CD11b^+^) through assessment of the kinetics of MHCII and PD-L1 (activation markers) expression at d 2, 7, 14, and 30 following both challenge and re-challenge *via* intracranial injection of AI9 peptide (Figure 2A). Among the animals that were subjected to challenge, a rapid upregulation of MHCII (74.2 ± 1.8% vs. 17.6 ± 2.9% without peptide) and PD-L1 (74.5 ± 2.8% vs. 13.3 ± 1.3% without peptide) was observed at d 2 (Figures 2B,C, Supplementary Figures 1C,D). However, over the time course of Ag exposure expression of these two activation markers waned i.e., MHCII was reduced to 30.8 ± 2.1% at d 7, 10.1 ± 1.0% at d 14 and 4.5 ± 0.5% at d 30 post-challenge (Figures 2B,C). Similarly, expression of PD-L1 decreased to 23.3 ± 1.8% at d 7, 10.5 ± 1.4% at d 14 and 4.2 ± 0.4% at d 30-post re-challenge (Figures 2D,E). In sharp contrast, subsequent re-challenge resulted in more modest upregulation of MHCII (45.7 ± 1.2%) and PD-L1 (34.6 ± 1.3%) at d 2-post re-challenge. To ensure the microglial cell inflammatory response detected was due to cognate peptide delivered into the CNS (and not due to the intracranial injection itself) we used control groups where saline was injected. Among control animals, MHCII expression was 17.6 ± 2.9% following challenge and its expression was 20 ± 1.4% following re-challenge; similarly, PD-L1 expression was 13.3 ± 1.3% and 20.2 ± 0.8% at d 2 post-challenge and re-challenge, respectively (Supplementary Figures 1A,C,D). These data demonstrated that microglial cells displayed prolonged activation in response to subsequent re-challenge when compared to challenge alone. Microglia displayed sustained, elevated MHCII expression (46.3 ± 1.0%) at d 7; d 14 (31.7 ± 2.1%), and d 30 post re-challenge (12.7 ± 1.0%). Similarly, a prolonged elevation of PD-L1 expression was also observed (48.8 ± 2.9%) at d 7; d 14 (31.5 ± 1.0%); and d 30 post re-challenge (12.9 ± 1.2%) when compared to challenge alone (Figures 2B–E).

### Re-challenge With T-Cell Epitope Peptide Results in Sustained Microglial Proliferation

**Figure 3:**
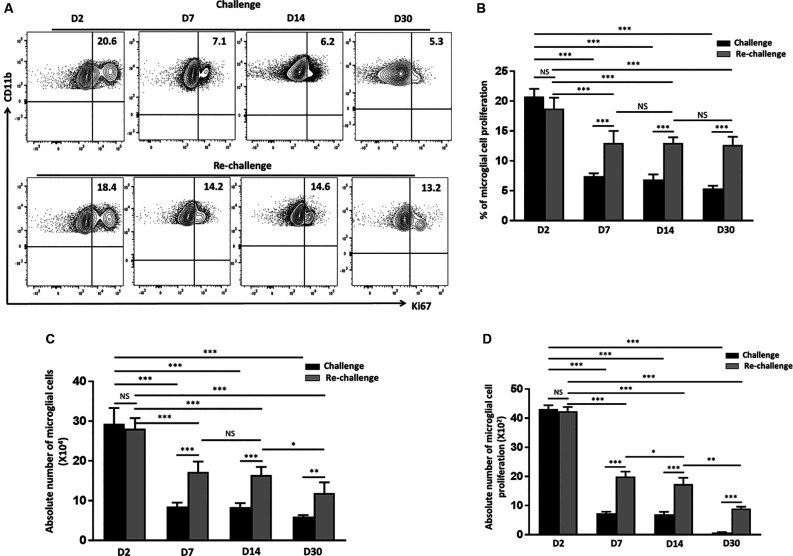
*In vivo* peptide re-challenge resulted in sustained microglial cell proliferation. **(A)** Representative contour plots present the expression of Ki67 on CD45^int^/CD11b^+^ microglial cells at d 2, 7, 14, and 30 post-challenge and re-challenge with cognate antigen. **(B)** Bar graph presents pooled data as a percentage (mean ± SD) of Ki67 expression on the microglial-gated population. **(C)** Bar graph presents the absolute numbers of microglial cells among challenged and re-challenged groups at d 2, d 7, d 14, and 30. **(D)** Pooled data show the absolute numbers of proliferating microglia among challenged and re-challenged groups at the indicated time points. Data are derived from two independent experiments using six animals/group/time point (two brains pooled together). *p < 0.05, **p < 0.01, ***p < 0.001; non-significant (NS) represent groups with no significant differences.

Pathological neuroinflammation generally displays a progressive expansion of the microglial cell population (Gomez-Nicola et al., 2013; Olmos-Alonso et al., 2016). In parallel to assessing their activation status, we went on to evaluate the microglial proliferative response to both challenge and re-challenge. To achieve this, BMNC were isolated at different time points from animals subjected to Ag challenge and re-challenge and stained for Ki67, a marker for proliferating cells. We then assessed the microglial expression of Ki67. At d 2 post-stimulation, the frequency of microglia expressing Ki67 was 20.7 ± 1.2% in the group receiving challenge; and 18.7 ± 1.8% in the animals receiving subsequent re-challenge, indicating an immediate response to specific Ag exposure in both the groups (Figures 3A,B). Further analysis of Ki67 expression showed a significant increase in the frequency of the proliferating microglia at d 7, d 14, and d 30 among the re-challenged animals when compared to the challenged animals. As shown in Figure 3, Ki67 expression in microglia from animals receiving re-challenge was 12.9 ± 2% at d 7, 13 ± 0.94% at d 14, and 12.6 ± 1.3% at d 30 post re-challenge. In contrast, microglial cells obtained from animals receiving challenge alone expressed Ki67 in 7.4 ± 0.4% at d 7, 6.8 ± 0.8% at d 14, and 5.3 ± 0.4% at d 30 post-challenge. This microglial cell population showed prolonged proliferative responses in the re-challenged group. We next evaluated the absolute number of microglia at all time points among the challenge and re-challenge groups. An increased number of microglial cells was observed in both challenged and re-challenged groups at d 2 (Figures 3A,C). However, among the challenged groups of animals, the number of microglial cells subsides to normal levels (Supplementary Figure 1B). In sharp contrast, the number of microglial cells remained elevated among the re-challenged group at all time points when compared to the challenged group (Figure 3C). Additionally, similar observations were made when the absolute number of proliferating microglia were evaluated (Figure 3D). These data demonstrate prolonged microglial cell expression of Ki67 in response to the repeated Ag exposure.

### Re-challenge With T-Cell Epitope Peptide Results in Sustained Production of iNOS by Activated Microglia

**Figure 4:**
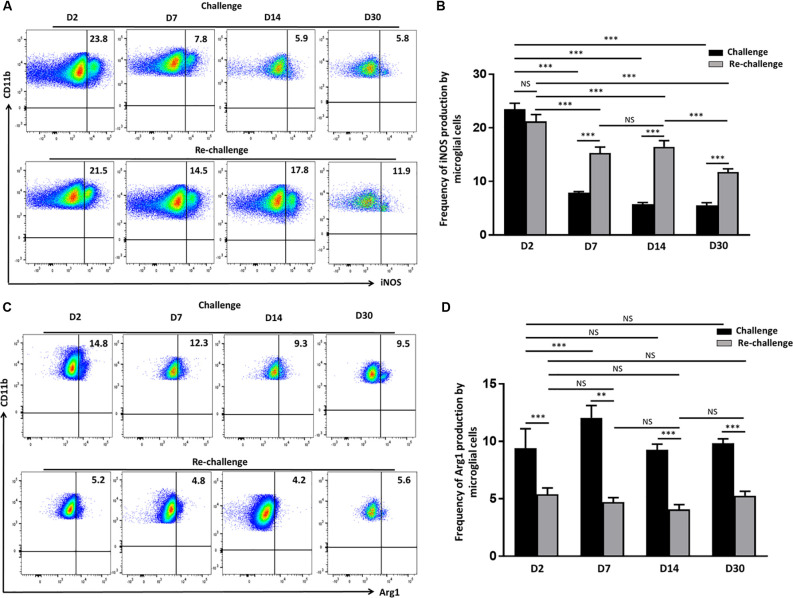
Re-challenge with T-cell epitope peptide-induced prolonged inducible nitric oxide synthase (iNOS) production by reactive microglia.** (A)** Representative pseudocolor plots illustrate microglial cell iNOS production at d 2, 7, 14, and 30 post-challenge and re-challenge with cognate antigen. **(B)** Bar graph indicates the frequencies of iNOS producing microglia under the indicated stimulation conditions. **(C)** Representative pseudocolor plot shows the percentage of the microglial cell expressing Arg1 in response to challenge and re-challenge at the indicated time points. **(D)** Bar graph presents pooled data showing the percentage cells expressing Arg1. Data presented as mean ± SD from two different experiments using six animals/group/time point (two brains pooled together). **p < 0.01, ***p < 0.001; non-significant (NS) represent groups with no significant differences.

Having observed that microglia displayed a long–term activated and proliferative phenotype in response to re-challenge, we then went on to assess if they also produced elevated levels of neurotoxic mediators that may be detrimental to neural tissue integrity. So, we evaluated the production of iNOS *via* intracellular staining among both challenge and re-challenge groups of animals at different time points post-stimulation. Elevated levels of iNOS were observed at d 2 both among the challenge (23.4 ± 1%) and re-challenge group (21.2 ± 1.2%) of animals (Figures 4A,B). Further analysis of iNOS production revealed that microglial cells from animals which were re-challenged produced it at a significantly higher frequency at d 7 (15.3 ± 1.0%); and persistent, elevated production was observed at d 14 (16.4 ± 1.1%), which remained higher at d 30 (11.7 ± 0.5%) when compared to the challenge groups (Figures 4A,B). Among challenged groups of animals iNOS production was 7.9 ± 0.2% at d 7, which further reduced to 5.7 ± 0.3% at d14, and 5.5 ± 0.4% at d 30. Finally, controls showed that administration of saline resulted in lower levels of iNOS production by microglia (Supplementary Figures 1A,B). To further investigate the effects of challenge and re-challenge on microglia, we evaluated the expression of anti-inflammatory marker arginase 1 (Arg1). Interestingly, we found that the expression of Arg1 was significantly reduced at all time points post re-challenge when compared to the challenged alone group of animals. The levels of Arg1 expression by microglia were 5.4 ± 0.5% at d 2, 4.7 ± 0.4% at d 7, 4 ± 0.4% at d 14, and 5.2 ± 0.3% at d 30 in the re-challenged group of animals. In contrast, among the challenged groups, the levels of Arg1 were 14.3 ± 0.5% at d 2, 12.4 ± 0.5% at d 7, 9.2 ± 0.4% at d 14, and 9.8 ± 0.4% at d 30 (Figures 4C,D). Taken together, these data indicate that there is increased iNOS and correspondingly decreased Arg1 production among microglia in animals receiving re-challenge with T-cell epitope peptide.

### Increased Production of Proinflammatory Mediators Following *Ex vivo* T-Cell Epitope Re-challenge

**Figure 5:**
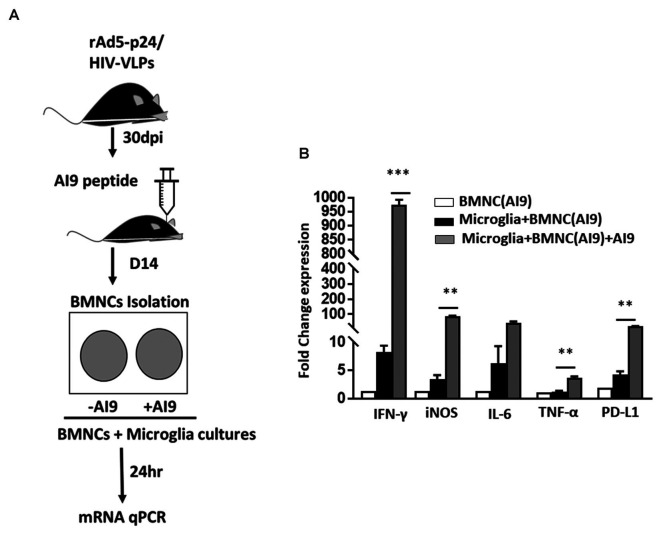
*Ex vivo* peptide re-challenge resulted in production of pro-inflammatory mediators. **(A)** Schematic of the experimental design shows that BMNC isolated at d 14 post-challenge were co-cultured with primary microglia. BMNC were either treated with HIV-specific AI9 peptide or left untreated before addition to the microglial cell culture. Real time RT-PCR was performed after 24 h. **(B)** Bar graph displays fold change in IFN-γ, iNOS, IL-6, TNF-α, and PD-L1 mRNA expression. Expression values were normalized to untreated controls. Data are presented as mean ± SD of two independent experiments using six animals. ***p < 0.001 and **p < 0.01.

Following our observation, that *in vivo* epitope peptide re-challenge resulted in prolonged microglial activation and sustained production of cytotoxic factors; we next investigated the impact of *ex vivo* re-challenge. In these experiments, BMNC which were isolated from animals at d 14 post-challenge were co-cultured with primary microglial cells, and re-challenged with AI9 peptide, or left unstimulated (Figure 5A). Using a semi-quantitative real-time (RT)-PCR we assessed the levels of IFN-γ, iNOS, IL-6, TNF-α, and PD-L1 transcripts, which demonstrate neuroinflammation. We detected significantly elevated IFN-γ and iNOS gene expression among the *ex vivo* AI9 re-challenged groups when compared to untreated controls. A reduced yet still significant upregulation of PD-L1 and TNF-α transcripts was also observed (Figure 5B). IL-6 transcript levels were also elevated compared to non-restimulated controls but did not reach statistical significance. We also evaluated the transcript levels of these markers among the BMNC control group. Data generated from these cells displayed negligible levels of IFN-γ, iNOS, IL-6, TNF-α, and PD-L1 transcripts.

## Discussion

The pathogenic potential of latent brain infection and the impact of periodic viral reactivation on the CNS is poorly understood. Accumulating studies have identified a link between viral re-challenge and neurodegeneration (Martin et al., 2014; Machado-Santos et al., 2018; Doll et al., 2020; Marcocci et al., 2020), however, the cellular events that contribute to it remain unclear. Elucidating the outcome of cumulative, repeated Ag challenge will be an important step towards understanding these mechanisms. Our previous findings report that Ag-specific bT_RM_ rapidly expand and produce inflammatory cytokines in response to their cognate Ag (Prasad et al., 2019). In this study, we provide further evidence that bT_RM_ cells also proliferate following repeated episodes of Ag exposure. In addition, a small fraction of these cells maintained homeostatic proliferation, as observed by Ki67 expression, and displayed increased survival in the absence of re-challenge. It has been reported that T_RM_ cells in the lung have limited longevity and that circulating T-effector memory cells which seed the lung help to maintain this stable population (Slutter et al., 2017). Although, bT_RM_ cells exhibit defects in survival capacity *ex vivo* (Wakim et al., 2010), these cells do possess high proliferative potential in response to reactivation events, which is similar to our finding (Steinbach et al., 2016).

The nature of viral infection shapes the phenotypic heterogeneity of CD8+ bT_RM_ cells. In this study, the frequency of CD69+CD103+CD8+ T-cells was around 80% following the Ag re-challenge, whereas only 25% of these cells persisted in the absence of Ag re-challenge. These data show that the chronicity of viral infection modulates the frequency of persisting bT_RM_ cells. Furthermore, among the saline group, we observed both CD103+ and CD103- populations. T_RM_ cells are a highly sensitive and activated T cell population and high numbers may not be required to persist in the absence of Ag, particularly in tissues like the brain. It is known that CD103-T-cells are recruited into the brain during the acute phase of infection but are not retained. There is also evidence, which indicates that CD103- populations can persist within the brain, albeit rather poorly when compared to the CD103+ counterparts. Genetic profiling of these cells indicates that CD103 expression influences T-cell retention, survival, and function within the CNS (Wakim et al., 2012). In our study, in analyzing the Ag-specific CD103- population among the saline group of animals, we observed lower expression of Bcl-2, which may indicate reduced survival of this CD103- population. However, further studies are required to address if the CD103- population upregulates CD103+ in response to Ag stimulation. A more detailed study is required to understand if Ag-specific cells get activated and expand in response to the peptide that drains into lymphoid tissue. However, it is clear from numerous reports that activated T_RM_ cells produce soluble mediators, which recruit additional T cells from the periphery (Tan et al., 2018; Wu et al., 2018).

IFN-γ is considered a major effector cytokine produced by T_RM_. Transcript levels of IFN-γ among *ex vivo* re-challenged groups of animals indicate immediate effector responses to Ag re-challenge. Our findings are in line with other studies where T_RM_ cells respond to previously encountered pathogens in an Ag-specific manner and express higher transcript levels of pro-inflammatory cytokines than their circulating counterparts (Hombrink et al., 2016; Kumar et al., 2017). The presence of preformed RNA encoding IFN-γ may also help explain the remarkable effectiveness of T_RM_ populations (Behr et al., 2018). While their survival and functional response does not wane over time, our data support the idea that the frequency of bT_RM_ cells declines in absence of cognate Ag. However, there are studies that report T_RM_ cells in the lung exhibit waning immunity (Slutter et al., 2017). While the protective role of T_RM_ to Ag re-challenge is well established, various aspects of bystander immune activation that may drive CNS injury remain unclear.

Brain-resident microglia detect and respond to pro-inflammatory triggers by switching to a reactive phenotype and releasing factors, which have neurotoxic side effects. This activation of microglial cells is a complex phenomenon and is associated with consequences ranging from neuroprotective to neurotoxic (Sierra et al., 2013), hence the critical need to understand these links. Microglia are a heterogeneous population whose phenotype depends on the local cytokine milieu, adopting various functions critical for neuronal health and CNS homeostasis (Perry et al., 2010). These cells normally express low levels of MHCII, however, under neuroinflammatory conditions activated microglia display highly upregulated expression (Schachtele et al., 2014).

The impact of repeated episodes of viral reactivation on microglial cell activation is unknown. In this study, we demonstrated that with repeated *in vivo* cognate peptide stimulation, microglial cells portray persistently activated profiles, as observed by MHCII and PD-L1 upregulation. Similarly, there are other studies, which report that MHCII signaling is critical for microglial cell activation, associated inflammatory responses, and neurodegeneration (Harms et al., 2013). Moreover, PD-L1 expression by microglia resulted in reduced cytotoxicity of cells expressing viral epitope peptides (Chauhan et al., 2021). PD-1: PD-L1 interactions are well-known to downregulate immune responses to prevent immunopathology. We have previously reported that T_RM_ cells within the brain express high levels of PD-1. Yet, despite this expression, bT_RM_ cells display increased cytokine production and proliferation in response to cognate antigen (i.e., these cells are not exhausted). These data indicate that PD-1 signaling is not only required to limit the severity of neuroinflammation but also functions to sustain the appropriate level of inflammation required to control the persistent or reactivated virus. In addition to an activated phenotype, we observed that microglia proliferate for an extended time in response to repeated Ag stimulation. Our findings are in line with others, which show that microglial cell expansion is a key component of neurodegenerative diseases (Gomez-Nicola et al., 2013).

IFN-γ receptors are abundantly and functionally present on microglia and chronic exposure to IFN-γ drives an exaggerated microglial response (Ta et al., 2019). Additionally, there are reports showing that exposure to recombinant IFN-γ is associated with microglial expansion, as well as NO release *via* iNOS (Papageorgiou et al., 2016). Similarly, in our study, prolonged activated microglial cells produced iNOS following repeated antigen exposure. Interestingly, these reactive microglia lacked expression of anti-inflammatory marker Arg1, indicating classical activation. Moreover, *ex vivo* peptide re-challenge resulted in increased transcript levels of cytokines such as IFN-γ, IL-6, and iNOS. Furthermore, there are reports which show that priming of microglia with IFN-γ not only induces cell proliferation and activation but also upregulates iNOS production, which may drive decreased neuronal information processing (Lynch, 2014; Ta et al., 2019). Importantly to our study, it is well established that neurons are sensitive to infection-induced-inflammatory stimuli, which may result in cellular damage. These findings indicate that sustained neuroinflammatory events may result in bystander CNS injury. Taken together, our findings indicate that repeated Ag-specific stimulation of adaptive immune responses leads to cumulative dysregulated microglial cell activation, proliferation, and production of neurotoxic mediators.

## Data Availability Statement

The original contributions presented in the study are included in the article/Supplementary Material, further inquiries can be directed to the corresponding author.

## Ethics Statement

The animal study was reviewed and approved by University of Minnesota Institutional Animal Care and Use Committee.

## Author Contributions

SP, SH, and JL conceived and designed the experiments and analyzed the data. SP, SH, WS, and PC performed experiments. All authors contributed to the article and approved the submitted version.

## Conflict of Interest

The authors declare that the research was conducted in the absence of any commercial or financial relationships that could be construed as a potential conflict of interest.

## Publisher’s Note

All claims expressed in this article are solely those of the authors and do not necessarily represent those of their affiliated organizations, or those of the publisher, the editors and the reviewers. Any product that may be evaluated in this article, or claim that may be made by its manufacturer, is not guaranteed or endorsed by the publisher.

## Supplementary Material

SUPPLEMENTARY FIGURE 1 Microglial cell activation status among saline controls. **(A)** Flow cytometry gating strategy for CD8+ T-cells and microglia. Microglial cells were identified as the CD45^int^CD11b^+^ population within total BMNC and leukocytes were identified within the CD45hi population. **(B)** Bar graph presents the absolute numbers of microglia among the challenged control group (saline-injected animals) at d 2, d 7, d 14, and d 30. **(C)** Representative contour images of the microglial population display the expression of activation markers (i.e., MHC-II and PD-L1) in control groups under the indicated stimulated conditions. **(D)** Bar graph shows frequencies of MHC-II and PD-L1 on microglial cells at d 2 following challenge and re-challenge of the saline controls. **(E)** Representative contour plots display proliferating microglial cells among the control animals at d 2 and d 14-post saline injection. **(F)** Bar graph shows the percentage of Ki67 expression by microglia among saline control groups at the indicated time points and among rAD-p24/HIV-VLP animals prior to saline injection. Pooled data are presented as mean ± SD from two independent experiments using six animals/group/time point.

SUPPLEMENTARY FIGURE 2 iNOS production by microglial cells among saline controls. **(A)** Representative pseudocolor plots showing the production of iNOS by the microglia among saline control animals at d 2 post-challenge and re-challenge. **(B)** Bar graph display pooled data of the frequency of iNOS production among the indicated treatment groups. **(C)** Contour plot displays the isotype control for Ki67 expression. **(D)** Contour plot shows Bcl-2 expression on the CD103- population pre-gated on Ag-specific CD8+ T-cells.
