# Supplementary figures and images for: Dysregulated Microglial Cell Activation and Proliferation Following Repeated Antigen Stimulation
Source: Front Cell Neurosci. 2021 Aug 10;15:686340. doi: 10.3389/fncel.2021.686340 (PMC8383069; doi:10.3389/fncel.2021.686340)

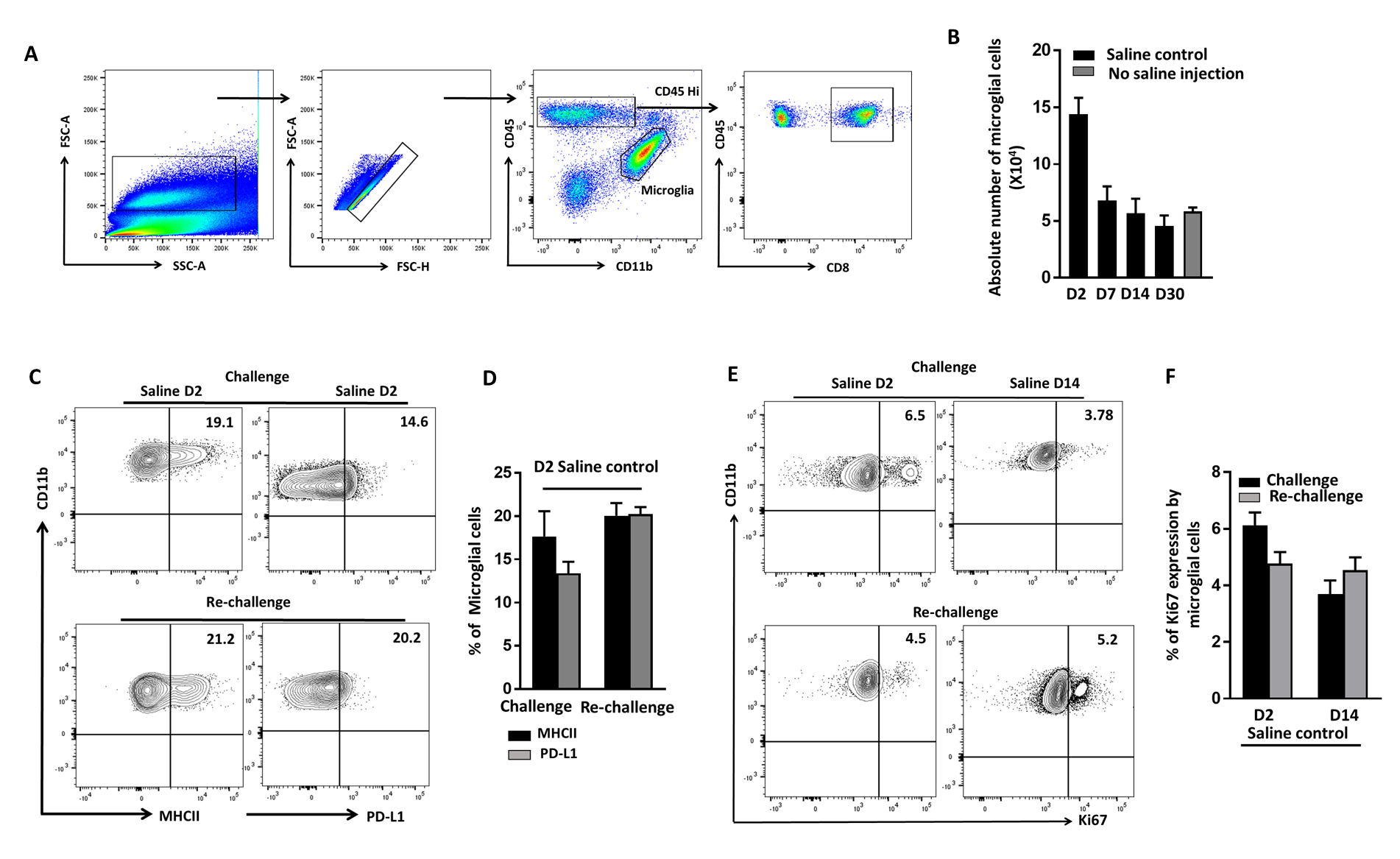

Supplement: SUPPLEMENTARY FIGURE 1 — Microglial cell activation status among saline controls. (A) Flow cytometry gating strategy for CD8+ T-cells and microglia. Microglial cells were identified as the CD45intCD11b+ population within total BMNC and leukocytes were identified within the CD45hi population. (B) Bar graph presents the absolute numbers of microglia among the challenged control group (saline-injected animals) at d 2, d 7, d 14, and d 30. (C) Representative contour images of the microglial population display the expression of activation markers (i.e., MHC-II and PD-L1) in control groups under the indicated stimulated conditions. (D) Bar graph shows frequencies of MHC-II and PD-L1 on microglial cells at d 2 following challenge and re-challenge of the saline controls. (E) Representative contour plots display proliferating microglial cells among the control animals at d 2 and d 14-post saline injection. (F) Bar graph shows the percentage of Ki67 expression by microglia among saline control groups at the indicated time points and among rAD-p24/HIV-VLP animals prior to saline injection. Pooled data are presented as mean ± SD from two independent experiments using six animals/group/time point. [file Image_1.TIF]

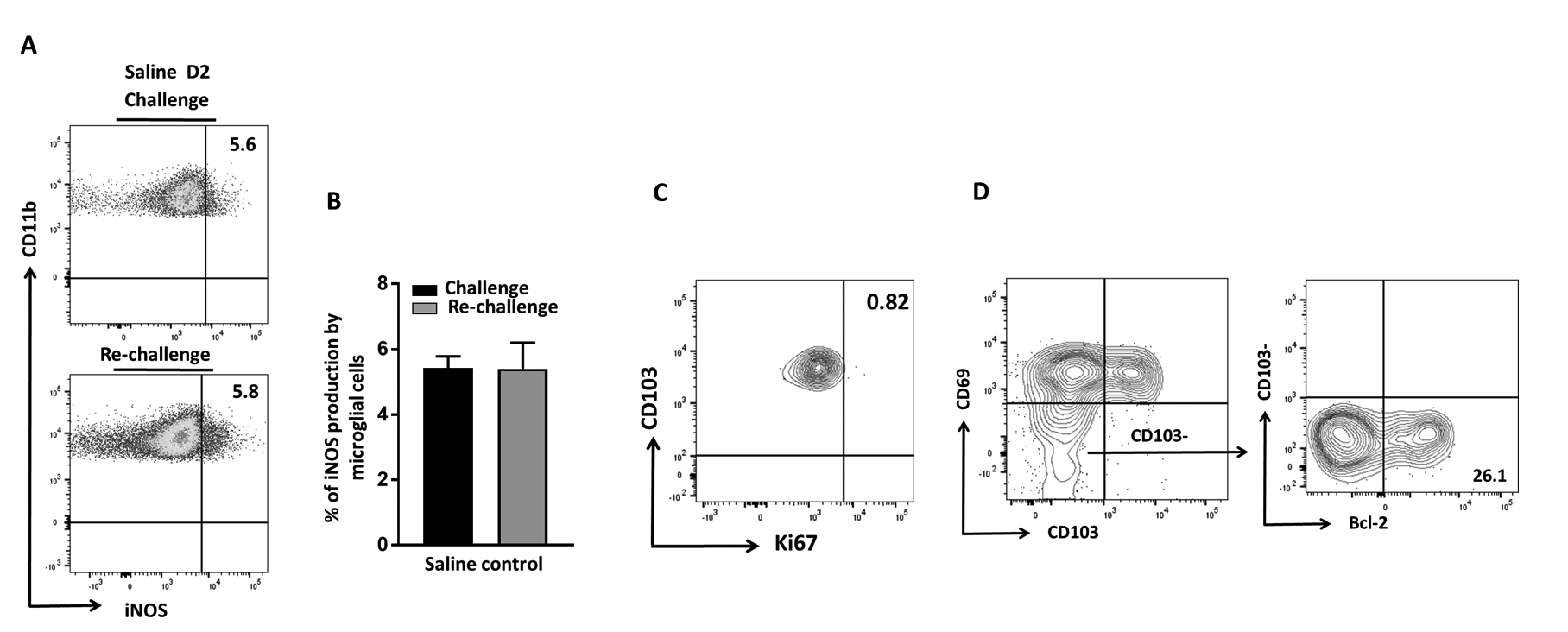

Supplement: SUPPLEMENTARY FIGURE 2 — iNOS production by microglial cells among saline controls. (A) Representative pseudocolor plots showing the production of iNOS by the microglia among saline control animals at d 2 post-challenge and re-challenge. (B) Bar graph display pooled data of the frequency of iNOS production among the indicated treatment groups. (C) Contour plot displays the isotype control for Ki67 expression. (D) Contour plot shows Bcl-2 expression on the CD103- population pre-gated on Ag-specific CD8+ T-cells. [file Image_2.TIF]
